# Association between hyperuricemia, prediabetes, and prehypertension in the Croatian adult population - a cross-sectional study

**DOI:** 10.1186/1471-2261-12-117

**Published:** 2012-12-04

**Authors:** Jasna Vučak, Milica Katić, Ivan Bielen, Davorka Vrdoljak, Dragica Ivezić Lalić, Ksenija Kranjčević, Biserka Bergman Marković

**Affiliations:** 1 Family Health Center, Ambulanta, Ulica XVIII Sukosan, Zadar, 23206, Croatia; 2 Department of Family Medicine, School of Medicine University of Zagreb, Zagreb, Croatia; 3 Department of neurology, Hospital “Sveti Duh”, Zagreb, Croatia; 4 Department of Family Medicine, School of Medicine University of Split, Split, Croatia; 5 Family Health Center, Novska, Croatia; 6 Family Health Center, DZ Zapad, Zagreb, Croatia

**Keywords:** Hyperuricemia, Prediabetes, Prehypertension, Purine-rich food, Prevalence

## Abstract

**Background:**

The association between hyperuricemia, hypertension, and diabetes has been proved to have strong association with the risk for cardiovascular diseases, but it is not clear whether hyperuricemia is related to the early stages of hypertension and diabetes. Therefore, in this study we investigated the association between hyperuricemia, prediabetes, and prehypertension in Croatian adults, as well as that between purine-rich diet and hyperuricemia, prediabetes, or prehypertension.

**Methods:**

A stratified random representative sample of 64 general practitioners (GP) was selected. Each GP systematically chose participants aged ≥ 40 year (up to 55 subjects) . Recruitment occurred between May and September 2008. The medical history, anthropometric, and laboratory measures were obtained for each participant.

**Results:**

59 physicians agreed to participate and recruited 2485 subjects (response rate 77%; average age (± standard deviation) 59.2 ±10.6; 61.9% women. In bivariate analysis we found a positive association between hyperuricemia and prediabetes (OR 1.66, 95% CI 1.09–2.53), but not for prehypertension (OR 1.68, 95% CI 0.76–3.72). After controlling for known confounders for cardiovascular disease (age, gender, body mass index, alcohol intake, diet, physical activity, waist to hip ratio, total cholesterol, low density lipoprotein, high density lipoprotein, and triglycerides), in multivariate analysis HU ceased to be an independent predictor(OR 1.33, CI 0.98–1.82, p = 0.069) for PreDM. An association between purine-rich food and hyperuricemia was found (p<0.001) and also for prediabetes (p=0.002), but not for prehypertension (p=0.41). The prevalence of hyperuricemia was 10.7% (15.4% male, 7.8% female), 32.5% for prediabetes (35.4% male, 30.8% female), and 26.6% for prehypertension (27.2% male, 26.2% female).

**Conclusion:**

Hyperuricemia seems to be associated with prediabetes but not with prehypertension. Both, hyperuricemia and prediabetes were associated with purine-rich food and patients need to be advised on appropriate diet.

**Trial registration:**

Current Controlled Trials ISRCTN31857696

## Background

A large number of studies have linked hyperuricemia (HU) with hypertension, cardiovascular disease, and diabetes [1,2]. An association was shown between elevated concentrations of serum uric acid (SUA) and increased mortality in patients with myocardial infarction, heart failure, stroke, and other diseases [3,4] and also with a purine-rich diet, alcohol intake, and a number of cardiovascular disease (CVD) risk factors [5].

Experimental models have demonstrated that an elevated concentration of serum uric acid (SUA) increases blood pressure without affecting the morphology of the kidney [6], and that lowering uric acid can normalize blood pressure [7]. In addition to the association between SUA and hypertension, many authors have confirmed the correlation between SUA and development of type 2 diabetes [8]. The results showed that every increase of serum uric acid by 59.5 μmol/L results in a 60% increase in risk for developing diabetes [9]. Although there is unquestionable evidence of the association between uric acid and oxidative stress, endothelial dysfunction, inflammation, subclinical atherosclerosis, and cardiovascular disease there is no consensus regarding its value as an independent risk factor [10,11].

Consequently, if hyperuricemia contributes to hypertension and diabetes, the assumption is that it should also contribute to prehypertension (PreHT) and prediabetes (PreDM). While hyperuricemia’s relationship with hypertension and diabetes has been well studied, few studies dealt with the relationship between hyperuricemia and PreHT [12,13], and PreDM [14]. The aim of our study is to assess whether SUA level is associated with PreHT and/or PreDM in an adult population in a Croatian primary care setting.

## Methods

### Study design

The survey was conducted between May 2008 and July 2008 within a representative, random sample of general practitioners (GP), who were enrolled by a random number generator based on a list of all registered family medicine practices (2374 in total) from the Croatian Institute for Health Insurance. Stratification of GP’s was made by region (coastal or continental), county (Croatia is divided into 21 counties), settlement size (up to 3999 inhabitants, 4000 to 9999, 10 000 to 29 999, 30 000 to 89 999, and 90 000 inhabitants and more), and number of insured patients based on a list of all GP’s/family medicine practices having a contract with the Croatian Institute for Health Insurance (national compulsory health insurance system covering 97% of the population) in 2007.

Taking into consideration the available data for the prevalence of risk factors for cardiovascular diseases in Croatia, and the expected dispersion of examined persons (20%), we determined that each GP should examine 55 patients (80% power, alpha 0.05, G*Power for Windows 3.1.3).

The research was carried out within the randomised clinical trial Cardiovascular Risk and Intervention Study in Croatia-family medicine (CRISIC- fm), and was registered as a clinical trial (Current Controlled Trials - ISRCTN31857696).

The study was approved by the Ethics Committee of Zagreb University School of medicine.

### Participants

Each general practitioner (GP) enrolled a consecutive, representative sample of participants aged ≥ 40 years (up to 55 subjects), who visited the practice from May 2008 to July 2008 who meet the criteria and provided informed consent to participate. The exclusion criteria included communication disability (dysphasia, aphasia), severe dementia or mental illness, and disease with an estimated life expectancy of less than six months.

All GP received precise instruction to enrol two patients per day between 10 am and 11am during their morning shift, and between 4 pm and 5 pm during their afternoon shift.

The interviews and two questionnaires that contained 140 and 127 items, respectively, were used to collect the socio-demographic, socio-economic, personal and family history data, and also information on the dietary and living habits, physical activity, medical therapy, psychological aspects and environment of the participants. The first questionnaire was administered and the anthropometric measurements were taken on the first day by the GP and the second questionnaire was given to patients to complete at home and return the following week when they arrive at the surgery for blood sampling after a minimum 8-hour fast.

### Measurement

All subjects were measured twice for height and weight (using identical standardised anthropometric scales), waist and hip circumference (by plastic coated, non-elastic centimetre tape), blood pressure (by mercury sphygmomanometer), and pulse rate. Data on smoking were obtained by self-assessment (smokers, former smokers who stopped smoking > 6 months, and nonsmokers). Moderate drinking was defined as 20 g ethanol per day for men and 10 g for women and the data were obtained by self-assessment. Overweight was defined as BMI ≥ 25 and obesity as BMI ≥ 30. Hypertension was defined as ≥ 140 systolic and/or ≥ 90 diastolic, or the use of antihypertensive drugs [15]. Diabetes mellitus was defined as fasting plasma glucose ≥ 7.0 mmol/L, or the use of hypoglycaemic drugs. Serum uric acid (SUA), fasting blood glucose (FBG), total cholesterol (TC), high density lipoprotein cholesterol (HDL), low density lipoprotein cholesterol (LDL), triglycerides (TG), creatinine, complete blood count, and complete urine analysis were performed. Hyperuricemia (HU) was defined as serum uric acid concentration ≥ 420 μmol/L in men and ≥ 360 μmol/L in women. PreHT was determined using JNC7 [16] definition, and considered to be blood pressure readings based on the average of 2 or more properly measured, seated BP readings on each of 2 or more office visits with a systolic pressure from 120 to 139 mm Hg or a diastolic pressure from 80 to 89 mm Hg. PreDM was determined using the criteria from the American Diabetes Association [17] with fasting plasma glucose level from 5.6 mmol/L to 6.9 mmol/L.

For dietary habits, the subjects were divided into 4 groups according to the frequency of purine-rich food consumption [18]. The first group comprised of subjects with a total of 0–5 points, the second 6–10 points, the third 11–15 points, and the fourth 16–20 points. The scoring is shown in Table 1.

**Table 1:** Scoring of purine-reach food consumption

	**Everyday**	**Few times a week but not everyday**	**Once a week**	**Once a month**	**Never or less than a month**	**Total**
Red meat and red meat products	4	3	2	1	0	
Wine and/or beer	4	3	2	1	0	
Liquor	4	3	2	1	0	
Sugar sweetened soft drinks (fructose)	4	3	2	1	0	
Animal fat	4	3	2	1	0	
Total						

### Statistical analysis

The basic characteristics of the sample were described by descriptive statistics. The differences between the categorical variables with relative risk for PreDM and PreHT were analyzed using the chi-square test. Logistic regression analysis was used to examine which variables were statistically significant risk factors in certain groups in relation to and adjusted for the relevant confounders using bivariate analysis. All values were interpreted according to a significance level of 95% (CI 95%, P < 0.05). All statistical methods were performed using SPSS for Windows (19.0.0.1, SPSS Inc., Chicago, Illinois, 2011).

## Results

Fifty-nine physicians recruited a total of 2485 subjects (response rate 77%). There were 61.9% women (mean age (± standard deviation) 58.9 ± 10.5) and 38.1% men (mean age 59.5 ± 10.7). Their basic characteristics are shown in Table 2.

**Table 2:** Basic characteristics of the study population

**Characteristic**	**Total**
**(mean ± SD); n (%)**	**N=2485**
Age(years)	59.2±10.6
BMI, kg/m^2^	28.9±4.9
Waist/hip ratio	0.9±0.1
Total cholesterol (TC) mmol/L	5.8±1.2
Low density lipoprotein cholesterol (LDL-C) mmol/L	3.5±1.08
High density lipoprotein cholesterol (HDL-C) mmol/L	1.5±0.4
Fasting blood glucose (FBG) mmol/L	6.0±2.3
Triglyceride (TG) mmol/L	1.9±1.4
Systolic blood pressure (SBP) mmHg	131.4±17.0
Diastolic blood pressure (DBP) mmHg	80.7±9.2
Serum uric acid (SUA) μmol/L	287.7±99.5
Smoking, n (%)	539 (22.4%)
Physically active, n (%)	1254 (56.0%)
Hyperuricemia, n (%)	240 (10.7%)
Prehypertension (PreHT), n (%)	595 (26.6%)
Prediabetes(PreDM), n (%)	729 (32.5%)
Alopurinol use, n (%)	64 (2.9%)

Smoking – actual smoking or less than 6 months since quitting.

Physically active – at least 150 min/week moderate to vigorous physical activity.

Hyperuricemia (≥ 420 μmol/L men, ≥ 360 μmol/L women).

Prehypertension PreHT (SBP 120–139 mmHg and/or DBP 80–89 mmHg).

Prediabetes PreDM (fasting blood glucose 5.6–6.9 mmol/L).

Hyperuricemia prevalence in the observed population was 10.7% (240) (men 15.4%, women 7.8%), and was more frequent in men (χ ^2^ = 33.6, p < 0.001). Although the prevalence of hyperuricemia was equal in men and women over 60 years of age, treatment with allopurinol was more frequent in men (χ ^2^ = 28.1, p<0.001).

The prevalence of PreHT was 26.6% (595) (men 27.2%, women 26.2) and of PreDM was 32.5% (729) (men 35.4%, women 30.8%).

The average values of SUA in men who had PreHT and PreDM did not differ relative to normotensive/non-diabetic man (t = 1.67, p = 0.097). In contrast, women with PreHT/PreDM have higher averaged values of SUA (t = 3.37, p = 0.001).

Bivariate analysis showed that hyperuricemia was significantly associated with PreDM (OR 1.71, CI 1.29–2.28, p < 0.001) but not with PreHT in men (OR 1.75, CI 0.70–2.39, p = 0.23) and women (OR 1.75, CI 0.66–2.62, p = 0.262). Only BMI was a predictor for PreHT. The data are shown in Table 3. All significant bivariate findings were used in multivariate logistic regression models for PreDM and PreHT. After controlling for the impact of significant risk factors (gender, BMI status, alcohol intake, diet, smoking, and triglycerides) HU ceased to be a significant independent predictor (OR 1.33, CI 0.98–1.82, p = 0.069) for PreDM . For the PreHT group, only non-smoking habit was a significant predictor when controlled for BMI status, smoking, and triglycerides (OR 1.52, CI 1.16–1.99, p = 0.03).

**Table 3:** Odds ratio of PreDM and PreHT based on clinical variables

**Variables**	**Prediabetes**			**Prehypertension**		
	**OR**	**95% CI**	**p**	**OR**	**95% CI**	**p**
Female gender	0.71	0.6–0.9	<0.001	0.79	0.63–1.03	0.059
Age ≥ 60 yrs	1.17	0.99–1.40	0.067	1.14	0.90–1.45	0.273
Smoking	0.72	0.57–0.90	0.005	0.65	0.50–0.85	0.002
Hyperuricemia (≥420 m i ≥360 f)	1.71	1.29–2.28	<0.001	1.34	0.87–2.14	0.180
BMI overweight (25–29.9 kg/m^2^)	1.41	1.11–1.79	0.005	1.38	1.05–1.80	0.020
BMI obese (≥30 kg/m^2^)	2.01	1.58–2.57	<0.001	1.34	0.99–1.79	0.060
Waist-to-hip ratio (WHR)	1.44	1.19–1.74	<0.001	1.16	0.91–1.47	0.245
Pro-uric diet (score >10)	1.61	1.25–2.06	<0.001	1.17	0.84–1.64	0.393
Alcohol consumption	1.3	1.06–1.58	<0.001	0.97	0.75–1.24	0.848
Triglycerides (>1.7mmol/L)	1.47	1.23–1.75	<0.001	1.27	1.01–1.60	0.045
Total cholesterol (>5.0 mmol/L)	1.09	0.86–1.30	0.560	0.87	0.67–1.14	0.365
HDL-cholesterol (m <1.03, f <1.29 mmol/L)	1.24	0.99–1.56	0.069	0.88	0.65–1.21	0.440
LDL-cholesterol (>3.0 mmol/L)	1.03	0.83–1.27	0.829	0.84	0.63–1.11	0.250

Smoking – actual smoking or less then 6months since quitting.

Waist-to-hip ratio – m >0.9, f >0.85.

Pro-uric diet – according to Table 1.

Alcohol consumption – > 20g ethanol per day for men and > 10g for women.

Participants with a higher pro-uric food intake were obese (*χ*^2^ = 14.01, p = 0.007) and had higher uric acid values (*χ*^2^ = 10.29, p = 0.006). Participants with PreHT do not show significant differences in the frequency of pro-uric food intake, while participants with PreDM consume pro-uric food more often as it shown in Table 4.

**Table 4:** Relationship between purine-rich diet and HU, PreHT, PreDM, and BMI

Diet*	**N (%)**	**HU**	**PreHTN**	**PreDM**	**BMI(25–29.9)**	**BMI(>30)**
		**N=228**	**N=641**	**N=771**	**N=994**	**N=834**
		**N (%)**	**N (%)**	**N (%)**	**N (%)**	**N (%)**
Score 0**–**4	873 (37.4%)	56 (24.6%)	223 (34.8%)	255 (33.1%)	373 (37.5%)	284 (34.1%)
Score 5**–**10	1156 (49.5%)	128 (56.1%)	327 (51.0%)	382 (49.5%)	490 (49.3%)	428 (51.3%)
Score 11**–**15	292 (12.5%)	43 (18.9%)	87 (13.6%)	127 (16.5%)	126 (12.7%)	116 (13.9%)
Score 16**–**20	13 (0.6%)	1 (0.4%)	4 (0.6%)	7 (0.9%)	5 (0.9%)	6 (0.7%)
P**		<0.001	0.406	0.002	0.200	0.009

*frequency of pro-uric food intake according to Table 1.

**X^2^ test.

## Discussion

This study showed an association between hyperuricemia and prediabetes, whereas the association with prehypertension was questionable and significant only when associated with increased BMI and triglycerides. Furthermore, we found that BMI remained the only predictor for prehypertension, while hyperuricemia was a predictor for prediabetes when combined with BMI. This is compatible with the study of Ishizaka et al. proposing a correlation between SUA, BMI, and waist circumference [19]. The PreCIS study [20] has shown that uric acid levels are increased in patients with hypertension and diabetes, while we showed that this association also exists in patients with prediabetes, even when other risk factors for cardiovascular disease are excluded.

There are a number of studies associating oxidative stress with the development of diabetes mellitus and its complications [21]. Since SUA is one of the most powerful water-soluble antioxidants, produced mainly by endothelial cells, there are speculations that high concentrations of SUA are the result of the body defensive mechanisms. On the other hand, norepinephrine infusions and/or angiotensin II lead to reversibly elevated blood pressure and SUA. In this context, a possible mechanism could be an increase in sympathetic activity leading to oxidative stress, and the body’s effort to reduce damage using its most powerful antioxidant and to the breakdown of compensatory mechanisms. This is supported by studies that associated increases in norepinephrine level, blood pressure, and SUA with increased body mass and further hypertension progression [22].

It is known that uric acid clearance is inversely correlated with insulin resistance, which is the main pathophysiological factor of all metabolic syndrome (MS) components. There is some level of agreement that uric acid levels should be determined in MS patients because of the association with major cardiovascular risk, especially in woman [23]. This study shows that other components of metabolic syndrome (BMI, TG, and WHR) also play a significant role in prediabetes incidence in the presence of HU. Accordingly, the measurement of SUA should be introduced as an additional indicator of poor prognosis in patients with metabolic syndrome, which is in accordance with recent studies [24,25].

We found that HU prevalence in Croatian adults was 10.7% (15.4% male, 7.8% female) and was lower than in other populations. This may be because the population only included subject of Caucasian origin. The lack of a relationship between hyperuricemia and prehypertension may be partly explained by the lower prevalence of hyperuricemia in the studied population than in other populations, where it ranges from 21.3% to 26.2% [26-28].

Sundström et al. [29] confirmed the independent effect of elevated uric acid levels on hypertension incidence and on its progression. Therefore a higher incidence of hyperuricemia is to be expected in prehypertensive patients, but our study did not show this. This implies that the progression from prehypertension to hypertension occurs through other mechanisms favored by the increased SUA or occurred in parallel with the increase, but is not caused by it. This conclusion is consistent with findings from a previous study by the same authors, who followed a subgroup for 12 years and observed the connection between uric acid levels and hypertension incidence disappeared. It is also in concordance with findings that allopurinol administration improves endothelial function but has no significant effect on systemic blood pressure in patients with type II diabetes and mild hypertension [30].

The prevalence of HU in women and men was similar in older age groups. Fang et al. found a strong relationship between increased UA and cardiovascular mortality among women [11], even after adjusting for diuretic use and menopausal status. Considering this fact, which was also demonstrated by our study, the relationship between HU and prehypertension is especially strong in postmenopausal women probably because they lose the uricosuric effect of estrogen. This may contribute to the increased risk of adverse cardiovascular events.

To the best of our knowledge, most previous studies have not been taken into account eating habits even though they could affect UA levels. We therefore sought to investigate further and divide the patients into 4 groups according to their dietary habits (consumption of purine rich-food). The obtained results continued to correlate HU with prehypertension and prediabetes in spite of differences in dietary habits.

### Strength and limitations of the study

Strength: The large community based sample and adjustment for numerous potential confounders, including eating habits, of the studied population strengthen our investigation.

Limitations: It was a cross-sectional study and so the causal relationship between serum uric acid concentration with prediabetes and prehypertension cannot be evaluated.

## Conclusion

Since prehypertension and prediabetes are pre-stages of hypertension and diabetes, respectively, it can be expected that HU will be more often present in persons suffering from prehypertension and/or prediabetes. We have proved that this is the case only with prediabetes and not with prehypertension. Purine-rich food is related with both, HU and prediabetes, and patients need to be advised on appropriate diet.

## Abbreviations

HU: Hyperuricemia; SUA: Serum Uric Acid; CVD: Carddiovascular Disease; PreHT: Prehypertension; PreDM: Prediabetes; BMI: Body Mass Index; WHR: Waist To Hip Ratio; TC: Total Cholesterol; LDL: Low Density Lipoprotein Cholesterol; HDL: High Density Lipoprotein Cholesterol; TG: Triglycerides; GP: General Practitioner; FBG: Fasting Blood Glucose.

## Competing interests

The authors have no conflict of interest to disclose related to this study.

## Authors’ contributions

JV researched the data and wrote the manuscript, MK reviewed/edited the manuscript, IB contributed to the discussion and edited the manuscript, KK contributed to the discussion and edited the manuscript, DV contributed to the discussion and edited the manuscript, DIL contributed to the discussion and edited the manuscript, and BBM reviewed/edited the manuscript. All authors read and approved the final manuscript.

## Pre-publication history

The pre-publication history for this paper can be accessed here:

http://www.biomedcentral.com/1471-2261/12/117/prepub
